# Regional Anesthesia Use in Pediatric Burn Surgery: A Descriptive Retrospective Series

**DOI:** 10.7759/cureus.19063

**Published:** 2021-10-26

**Authors:** Michael Richman, Jeffrey M Berman, Elizabeth M Ross

**Affiliations:** 1 Department of Pediatric Anesthesiology, Seattle Children's Hospital, Seattle, USA; 2 Department of Anesthesiology, University of North Carolina at Chapel Hill, Chapel Hill, USA

**Keywords:** opioid sparing, pediatric burn pain management, burn grafting, pain management, burn pain, regional anesthesia, pediatric anesthesia

## Abstract

Pediatric burns are a common and often devastating injury. Treatment, reconstruction, and rehabilitation are painful experiences. For some, the experience triggers post-traumatic stress disorder and/or a chronic pain syndrome. Given the role pain plays as a major secondary disease, it must be addressed to achieve optimal healing. Regional anesthesia has been used extensively to manage postoperative pain and reduce the need for opioids following other surgical procedures in children. However, regional anesthesia is not widely used in pediatric burn care. We present a descriptive, retrospective case series of 15 pediatric burn patients who received regional anesthesia as part of their intra-operative pain management. In our patient population, we saw low levels of anesthetic as well as opioid usage with well-controlled pain. In this cohort, 93% of patients scored a 0/10 on the Face, Legs, Activity, Cry and Consolability (FLACC) scale for pain by post-anesthesia care unit (PACU) discharge, with an average PACU stay of 70 minutes. Thirty-three percent of patients received no opioids, with the average opioid dose being 0.06mg/kg morphine equivalents. This case series serves to make clinicians aware of the feasibility of use and benefits of regional anesthesia in pediatric burn patients requiring operative repair.

## Introduction

Burn injuries are the most common injury to pediatric patients in the world, accounting for approximately 2500 fatalities annually in the United States [1]. Of those reported, scald burns are the main culprit in children younger than five years of age, while flame-related burns are more commonly associated with older children and adolescents [2]. Burns are traumatic both physically and emotionally, especially to vulnerable children. Serial dressing changes, as well as physical and occupational therapy, cause repetitive trauma, particularly when pain is not well controlled early in treatment [3]. Poorly controlled pain leads to decreased compliance with treatment regimens and can delay and degrade wound healing through altered neurohormonal responses. Wollgarten-Hadamek demonstrated that children aged nine to sixteen years who had suffered moderate to severe burns at age six to twenty-four months had long-term alterations in somatosensory and pain processing [4].

Physicians have historically used opioids to manage pain from burn injuries. Even when multi-modal analgesics such as acetaminophen, non-steroidal anti-inflammatory medications, and gabapentin are used, optimal pain control is often not achievable [3]. One of the most painful portions of burn treatment is the initial operative repair. Regional anesthetic techniques can greatly decrease the pain levels experienced during this time, and it is well documented that regional anesthesia reduces opioid use [5]. Shank et al. found that children undergoing skin graft harvests who received nerve blocks at the donor site had less postoperative pain than those receiving surgical infiltration of local anesthetics [6]. Rustad et al. demonstrated adult burn patients receiving peripheral nerve catheters required significantly fewer analgesics in the 48 hours after the nerve catheter was placed [7]. Similarly, Cordts et al. detailed a 77% reduction in morphine requirement in adult burn patients along with decreased reported pain levels after administration of single-shot or continuous catheter local anesthesia [8]. Though Richards et al. demonstrated a downward trend in opioid analgesia in the pediatric burn population between 2013 and 2018 [9], the benefits of regional anesthesia have not yet been widely reported in this patient population [10].

The benefits of this decreased opioid use are multifold: improved bowel function, decreased potential for short- and long-term opioid sensitization, decreased need for weaning and dependence, and reduced potential for long-term psychological effects. In addition to analgesia, the sympathectomy-induced vasodilation of local anesthetics increases tissue blood flow and thus possibly improves local wound healing. Though little literature exists specific to burn healing, it has been shown that regional anesthesia decreases the failure rate of arteriovenous fistulas via a similar mechanism [11].

The aim of this case series is to make clinicians aware of the feasibility of use and benefits of regional anesthesia in pediatric burn patients requiring operative repair. This article was previously presented as a meeting abstract at the 2020 Society of Pediatric Anesthesia annual meeting on February 29, 2020. This article was previously posted to the medRxiv preprint server on November 25, 2020.

## Materials and methods

The study occurred at a university tertiary health care system that is an American Burn Association verified burn center. The patient database from the acute pediatric pain and regional service, which contains all regional blocks performed as well as any complications found, was manually examined by two people from February 2019 to October 2019. Anesthetic records of all patients under 18 years of age who received a regional anesthetic block for operative repair of burn injuries were identified. We then analyzed the perioperative period of these patients to determine the type of regional block, type and dose of local anesthetic used, intra-operative analgesic and anesthetic usage, anesthesia time, as well as immediate post-operative analgesic usage, pain levels, and time spent in the post-anesthesia care unit (PACU). We have no interests to declare. The IRB determined that this study did not require consent other than that for the anesthetics performed.

## Results

We identified 15 patients aged seven months to 14 years who received general anesthesia and an appropriate regional block to cover both graft and donor sites for surgical burn debridement and grafting. Based on the American Society of Anesthesiologists (ASA) criteria, all patients were classified as ASA I or II. All regional blocks were performed after induction of anesthesia but prior to surgical incision. No complications with regional anesthesia were noted. All patients except one received up to 2.5 mg/kg of Bupivacaine 0.25% with or without clonidine depending on the block type; the other received 2.5 mg/kg of 0.5% Ropivacaine. All patients except one received approximately half the minimum alveolar concentration (MAC) of sevoflurane for the duration of their anesthetic. The only opioid given intra-operatively was fentanyl. Overall average intraoperative fentanyl dosing was 1.2 mcg/kg, with most patients receiving 0.5-0.9 mcg/kg. In addition, 9/15 patients received dexmedetomidine (average dose 0.6 mcg/kg) and 3/15 ketamine (average dose 0.75 mg/kg). Four patients received acetaminophen at either 10 mg/kg or 15 mg/kg based on their age. Two patients received 0.5 mg/kg of ketorolac. One-third of patients received no analgesic adjuvants in addition to the regional block and opioid. 

When examining pain scores and PACU stay, we found the following. Pain scores were recorded using the Face, Legs, Activity, Cry, Consolability (FLACC) scale, a behavioral pain assessment scale used for patients who are unable to self-report. Scores range from zero to ten, with zero being no pain. Sixty-seven percent of patients had FLACC scores of 0 upon arrival to the PACU. This rose to 93% by discharge, with an average discharge time of 70 minutes (range 33-158 minutes). Average opioid use in the PACU ranged from 0-0.2 mg/kg morphine equivalents with a mean of 0.06 mg/kg. One-third of patients received no opioids in the PACU, even after patients had recovered from anesthesia and met discharge criteria. Table 1 shows intraoperative anesthesia time, fentanyl use, PACU times, and FLACC scores, and PACU morphine equivalents given based on age. Table 2 stratifies these same criteria in terms of block(s) used. Twelve patients received fentanyl only at induction of anesthesia. The average PACU morphine equivalent for patients receiving fentanyl only at induction of anesthesia was 0.04 mg/kg. Three patients received additional fentanyl during the surgical procedure for an average of 2.5 mcg/kg of fentanyl total throughout the anesthesia time. The average PACU morphine equivalent for patients receiving fentanyl after induction of anesthesia but prior to the PACU was 0.1 mg/kg. 

**Table 1:** Perioperative opioid use, pain scores, anesthesia, and PACU times stratified by age PACU = post-anesthesia care unit, avg = average, mcg = micrograms, kg = kilograms, mg = milligrams, FLACC = Face, Legs, Activity, Cry, Consolability scale

Age range	Number of patients	Anesthesia time minutes range (avg)	Intraoperative fentanyl range (avg) mcg/kg	PACU time minutes range (avg)	PACU FLACC 0 minutes range (avg)	PACU FLACC 15 minutes range (avg)	PACU FLACC discharge range (avg)	PACU morphine equivalent range (avg) mg/kg
7 to 13 months	3	74-129 (109)	0-1.9(0.6)	33-69(53)	0	0-5(2)	0	0-0.03(0.01)
2 to 4 years	4	53-230 (115)	0.5-0.9(0.8)	44-53(48)	0	0-8(4)	0	0.01(0.03)
5 to 8 years	4	76-291 (166)	0.5-2.8(1.8)	44-158(90)	0-10(5)	0-6(1.5)	0	0.1-0.2(0.1)
11 to 14 years	4	65-197 (117)	0.5-1.7(1.2)	55-100(79)	0-7(2.5)	0-9(5)	0-5(1)	0.04-0.15(0.09)

**Table 2:** Perioperative opioid use, pain scores, anesthesia, and PACU times stratified by regional anesthetic block type PACU = post-anesthesia care unit, FLACC = FLACC = Face, Legs, Activity, Cry, Consolability scale, avg = average, mg = milligram, kg = kilogram *One patient received both supraclavicular and caudal blocks.  **Transversus abdominus Plane ***One patient received both femoral and popliteal blocks.

Block	Number of patients	Anesthesia time minutes range (avg)	Intraoperative fentanyl range (avg) mcg/kg	PACU time minutes range (avg)	PACU FLACC 0 minutes range (avg)	PACU FLACC 15 minutes range (avg)	PACU FLACC discharge range (avg)	PACU morphine equivalent mg/kg range(avg)
Supraclavicular*	4	74-129 (101)	0-1.9 (0.8)	33-69 (51)	0	0-5 (1)	0	0-0.03 (0.01)
TAP**	2	53-197 (125)	0.5-1.7 (1.1)	46-100 (73)	0-7 (4)	0-4 (2)	0-5 (2.5)	0-0.09 (0.05)
Femoral***	4	61-165 (106)	0.5-2.7 (1.2)	46-158 (96)	0-8 (4)	0-6 (4)	0	0-0.16 (0.08)
Popliteal***	2	77-132 (105)	0.5-2.7 (1.6)	46-75 (61)	0	0	0	0.1-0.15 (0.13)
Caudal*	2	116-124 (120)	0.9-1.9 (1.4)	53-58 (56)	0	0-8 (4)	0	0-0.03 (0.02)
Epidural	3	127-291 (216)	0.9-2.8 (1.8)	44-114 (71)	0-10 (4)	0-9 (6)	0	0.1-0.2 (0.12)

## Discussion

Undertreated acute pain can lead to chronic pain and psychiatric maladies such as post-traumatic stress disorder [3]. Pediatric burn patients are particularly susceptible to long-term suffering. Multiple previous studies examining other surgeries have demonstrated the benefits of regional anesthesia in the pediatric population with regards to pain control and decreased anesthetic usage [12]. In addition, patients have been shown to experience decreased urinary retention and post-operative nausea/vomiting as well as quicker discharge, return of ambulation, and overall recovery [13]. Increased use of perioperative opioids has been shown to lead to greater continued use of opioids [14]. Additionally, concern has been raised that the use of narcotic medications may potentially lead to adverse cognitive and psychomotor effects given the many opioid receptors in brain regions that moderate or modulate attention, memory, and learning tasks [15]. It can be concluded that decreased perioperative opioid utilization would prevent the physical and social consequences of abuse. Little data exists on regional anesthetic block use in pediatric burn injury patients. In our patient population, we saw that the use of regional anesthesia resulted in low post-operative FLACC scores with minimal to no opioid use in the perioperative setting. In addition, patients were able to receive less inhaled anesthetic, which reduced hemodynamic fluctuations from sympathetic activation.

One major benefit highlighted in studies on regional anesthetic block use is decreased PACU length of stay [12]. Shorter PACU times decrease hospital costs and improve operating room flow. While this series did not compare patient populations, our study population did experience objectively short PACU times, leading to an obvious benefit for the hospital as well as for the patient.

Insufficient literature exists describing regional anesthesia use in pediatric burn patients. This case series serves as a model and demonstrates the feasibility of regional anesthetic block use in pediatric patients requiring operative repair of burn injuries. In our patient population, we achieved low FLACC scores with minimal opioid use and no complications from the regional blocks.

This series is limited by its retrospective, descriptive nature. As such, patients received a variety of adjuvant pain medications depending upon the anesthesia care team and post-operative nurse. One patient received the highest post-operative opioid consumption despite FLACC scores of zero. This was likely attributed to individual nurse familiarity with regional blocks and could be alleviated with time and education. Future prospective studies comparing matched cohorts with and without blocks would further stratify the benefits of regional anesthesia in pediatric burn patients.

Another important limitation of this study was the focus on the immediate perioperative period. Burn injury has multiple points of insult that include the initial burn as well as subsequent dressing changes. In addition to the initial injury, repetitive insults from treatments and dressing changes further traumatize children. Indeed, many children view dressing changes as the most traumatizing aspect of their burn injury [16]. Children lack the ability to understand the long-term benefits of painful procedures and treatments; consequently, they are more susceptible to ensuant psychological maladies such as anxiety, depression, and post-traumatic stress disorder [13]. Though preventing pain in the immediate post-operative period may decrease these outcomes [17], prolonging this effect would provide even more benefit. Local anesthetic regional blocks often lend themselves to catheter placement which would lengthen the benefits seen in this series. Further studies looking at the feasibility of placement given burn location, effects on mobilization, healing, and opioid usage could improve outcomes in this patient population.

## Conclusions

Pain from burns can be excruciating and difficult to control. Excellent intraoperative and post-operative control can decrease experienced pain and suffering throughout the course of treatment. We presented a 15 patient case series of pediatric burn patients requiring operative grafting and debridement who received both general and regional anesthesia. Our patient population demonstrated excellent pain control with minimal opioid use. Clinicians should be aware of the feasibility and benefit of regional anesthesia in this patient population. Burn surgeons should discuss with anesthesiologists the possibility of regional anesthesia in their pediatric burn patients. The discussion of catheter placement for longer-term pain control should be visited as well.
